# Supplementary material for: Life and death in Trypillia times: Interdisciplinary analyses of the unique human remains from the settlement of Kosenivka, Ukraine (3700–3600 BCE)
Source: PLoS One. 2024 Dec 11;19(12):e0289769. doi: 10.1371/journal.pone.0289769 (PMC11633957; doi:10.1371/journal.pone.0289769)

Figure 16.1-4: MNI and skeletal representation: Bone inventory.

INDIVIDUAL 1: square B2-3, house center; calcined bones, F > M, adult.

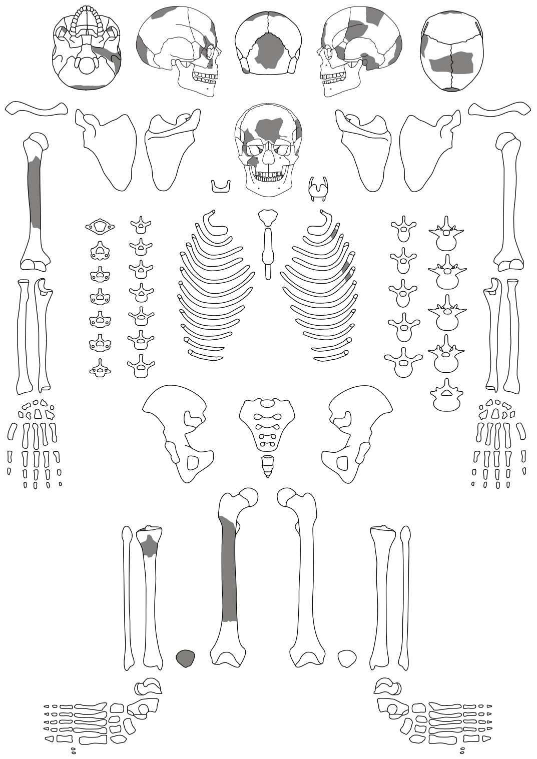

INDIVIDUAL 4: square Д3, eastern house; calcined bones, late juvenile/early adult female, 3942-3644 cal BCE (2σ, femur).

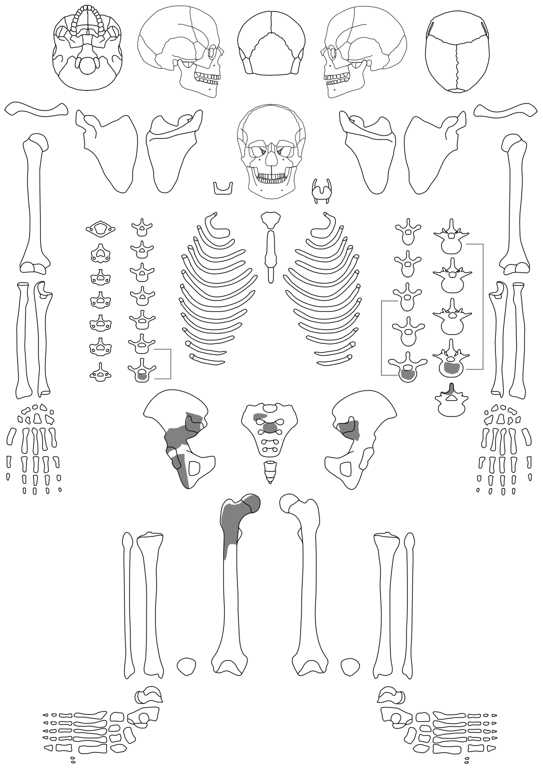

INDIVIDUAL 2: square B2-3, house center; calcined bones, younger child, 4240-3987 cal BCE (2σ, temporal).

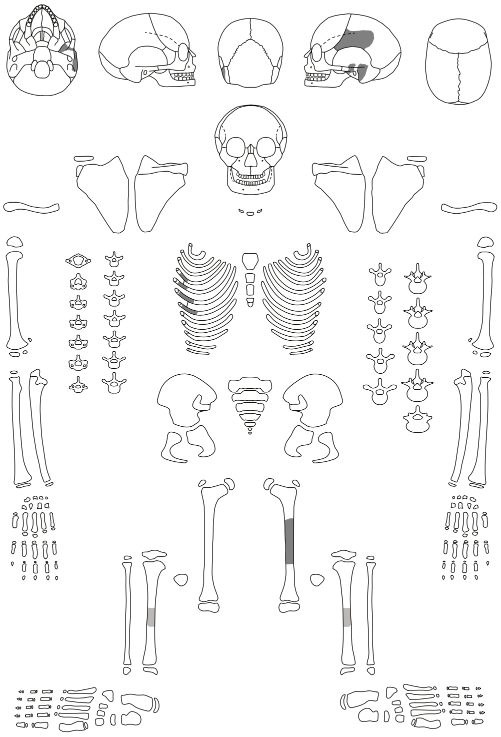

INDIVIDUAL 3: square B2-3, house center; calcined bones, older child.

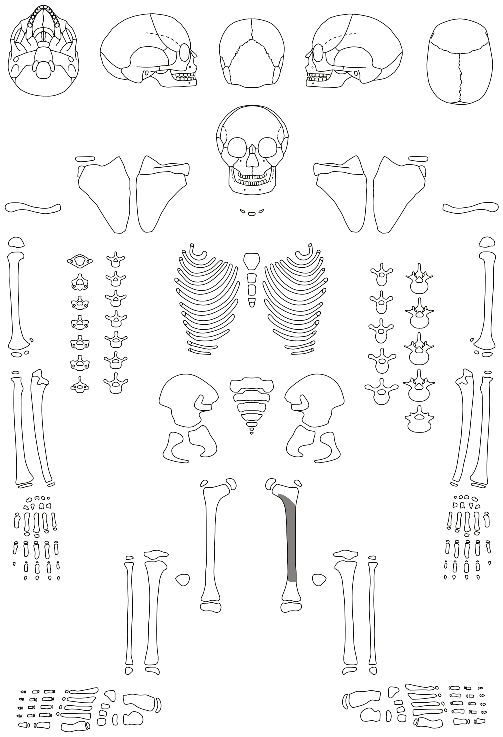

Figure 16.1-8: MNI and skeletal representation: Bone inventory.

INDIVIDUAL 5: square E4, eastern house periphery; unburnt bones, adult, 3766-3593 cal BCE (2σ).

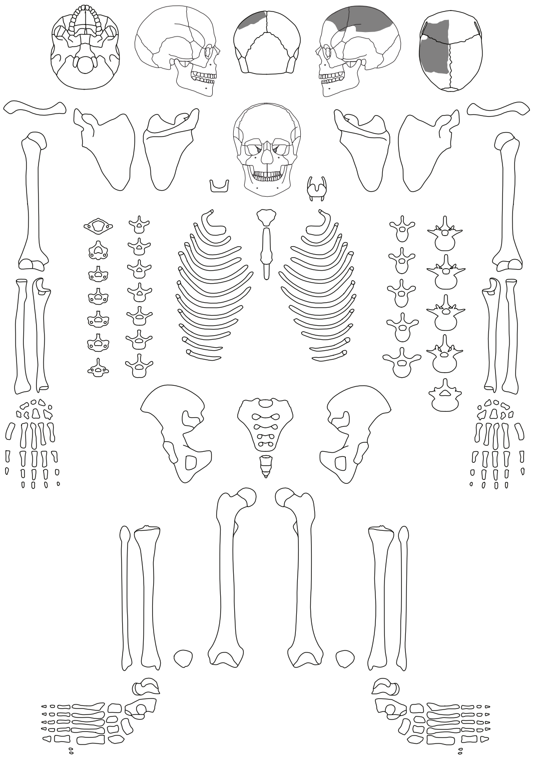

INDIVIDUAL 6: square Г5, southeastern house periphery; unburnt bones, adult, 3760-3532 cal BCE (2σ).

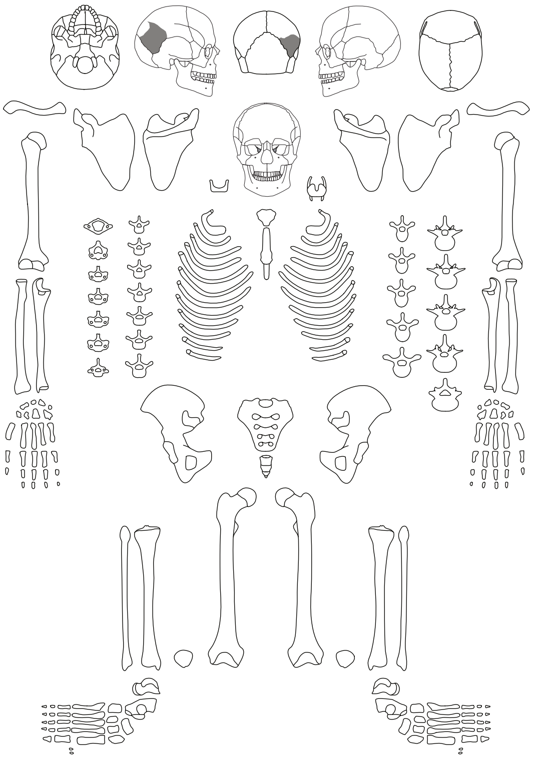

INDIVIDUAL 7: square B2 (upper layer),eastern house periphery; unburnt bones, adult, 3630-3374 cal BCE (2σ).

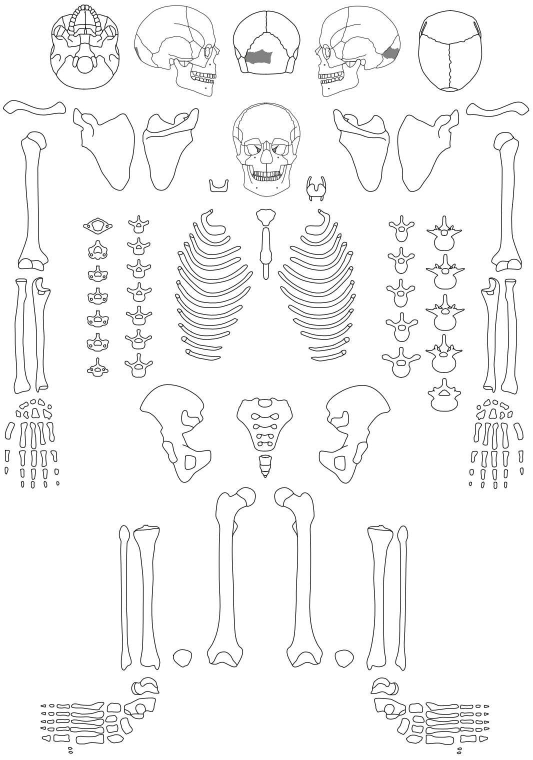

ISOLATED BONES (5,6,++), different contexts; unburnt, all adult. Indicated are ^{14}C dates (cal BCE, 2σ) and locality (sq.).

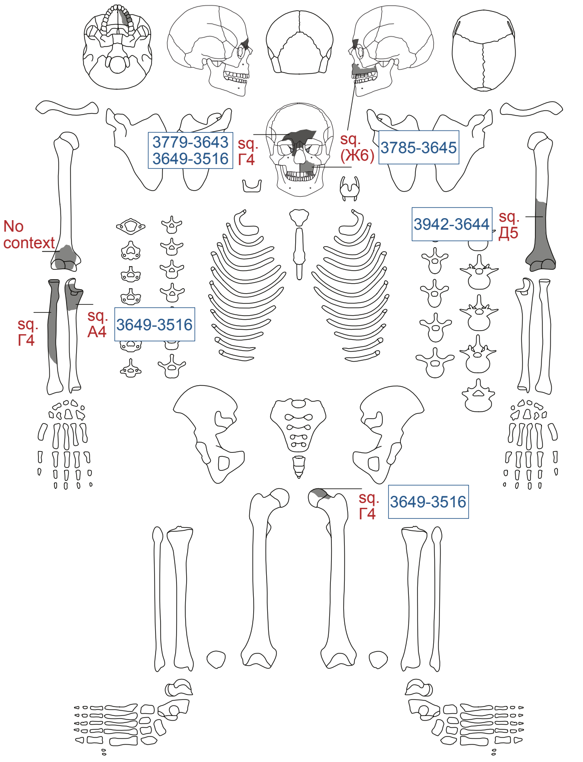

Figure 17.1-7: Histomorphology: age indicators, fire impact, and bioerosion.

For methods, see original article. For terms and descriptions of microbial attack see Hackett 1981, Microscopical Focal Destruction (Tunnels) in Exhumed Human Bones. *Medicine, Science and the Law* 21 (4), 1981, 243-265. **Micrographs:** K. Fuchs.

INDIVIDUAL 1, calcined remains. A: right humerus, middle diaphysis, transversal section, plain light, 5.6x magnification. **B**: right femur, proximal diaphysis, transversal section, polarized light, 20x magnification. **C**: Left parietal bone, vertical section, cortical bone of the lamina interna, plain light, 10x magnification.

Histomorphological features: OHI of 5, well-defined bone microstructure of a compact bone (Haversian canals, circumferential and interstitial lamellae, osteocyte lacunae with canaliculi); preservation of collagen structures (A-B), postmortem damage on periosteal and endosteal bone surface (A, green arrows). The high number of osteons of different generations (A, C, white arrows) indicate an adult age-at-death.

Fire impact: Colour; trapped carbon, burst osteons, heat cracks (black arrows).

Bioerosion: No signs of microbial destruction of bone tissue.

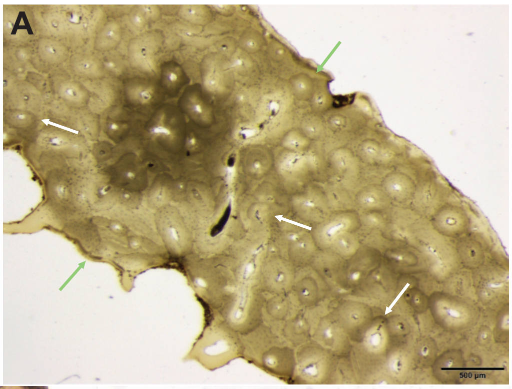

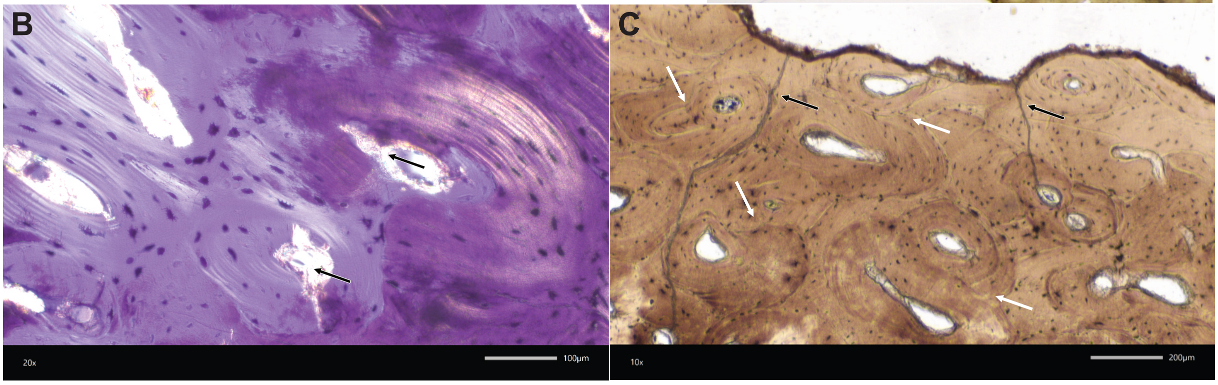

INDIVIDUAL 2, calcined remains. A-C: left femur, middle diaphysis, transversal section. **A**: Plain light, 5.6x magnification. **B**: Periosteal and inner part of the compact bone, polarized light, x10 magnification. **C**: Inner and endosteal part, plain light, x10 magnification.

Histomorphological features: OHI of 5, well-defined bone microstructure (see individual 1). Preservation of collagen structures, postmortem damage on periosteal and endosteal bone surface (A-C, green arrows). Ratio of lamellar bone (B, white arrows) to the number of (secondary) osteons (C, white arrows) is are typical for a bone in appositional growth, indicating the subadult age-at-death.

Fire impact: Colour; trapped carbon (often within the osteon circumferential lamellae; C, black arrows), burst osteons (A, black arrows).

Bioerosion: No signs of microbial destruction of bone tissue.

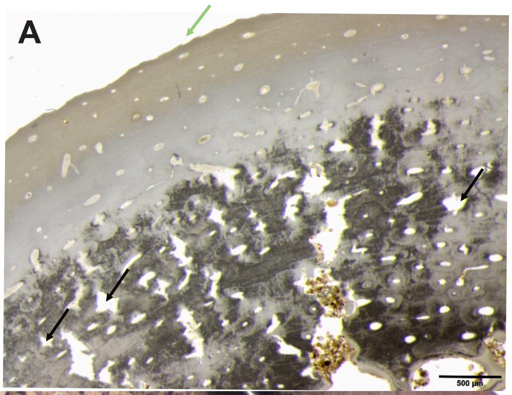

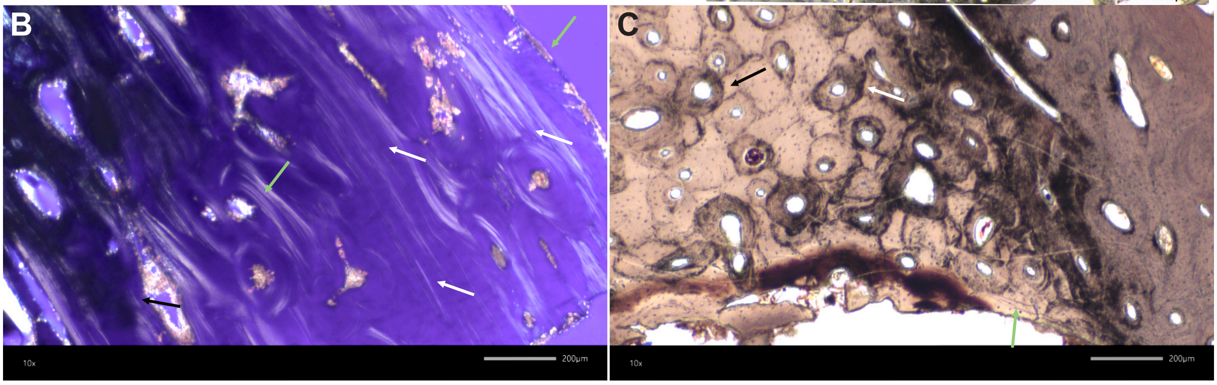

Figure 17.3-4: Histomorphology: age indicators, fire impact, and bioerosion.

INDIVIDUAL 3, calcined remains. A-C: left femur, middle diaphysis, transversal section. **A**: Plain light, 5.6x magnification. **B**: Inner part of the compact bone, polarized light, x10 magnification. **C**: As B, but plain light, x10 magnification.

Histomorphological features: OHI of 5, well-defined bone microstructure (see individual 1). Low preservation of collagen structures, postmortem damage on periosteal and endosteal bone surface (A, green arrows). Ratio of lamellar bone (B, white arrows) to the number of (secondary) osteons (C, white arrows) is similar to individual 2, but more osteons indicate an older age-at-death.

Fire impact: Colour; trapped carbon (B-C, black arrows), burst osteons (A, C, black arrows).

Bioerosion: No signs of microbial destruction of bone tissue.

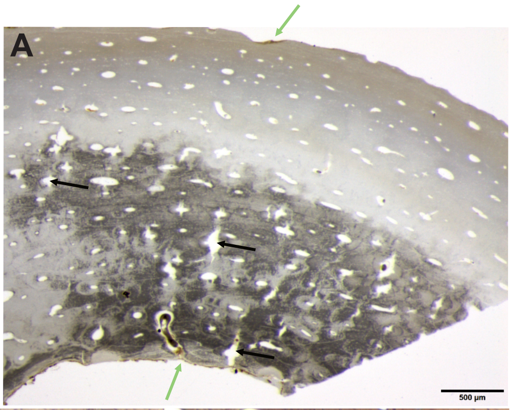

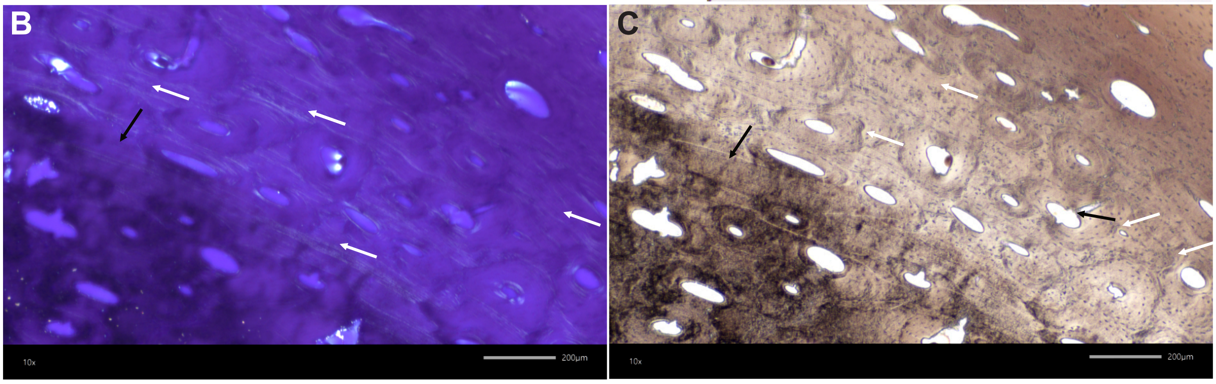

INDIVIDUAL 4, calcined remains. A-C: right femur, proximal-lateral diaphysis, transversal section. **A**: Plain light, 5.6x magnification. **B**: Inner part of the compact bone, polarized light, x10 magnification. **C**: Inner and endosteal part, plain light, x10 magnification.

Histomorphological features: OHI of 5, well-defined bone microstructure (see individual 1). Postmortem damage on periosteal and endosteal bone surface (A, C, green arrows). Patches of lamellar bone (B, white arrows) and osteons of different generations (B, black arrows) indicate non-mature bone and age-at-death, younger than individual 1, but older than individual 3.

Fire impact: Colour; trapped carbon (often within the osteon circumferential lamellae; C, black arrows), burst osteons (A, C, black arrows).

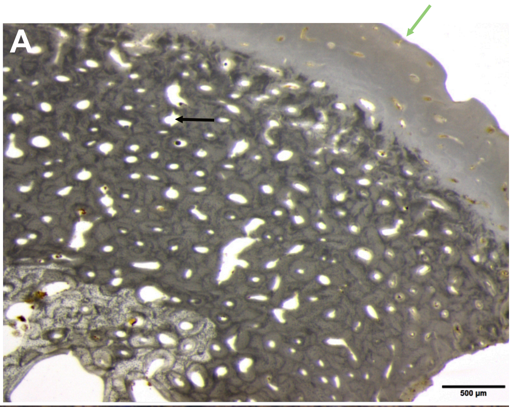

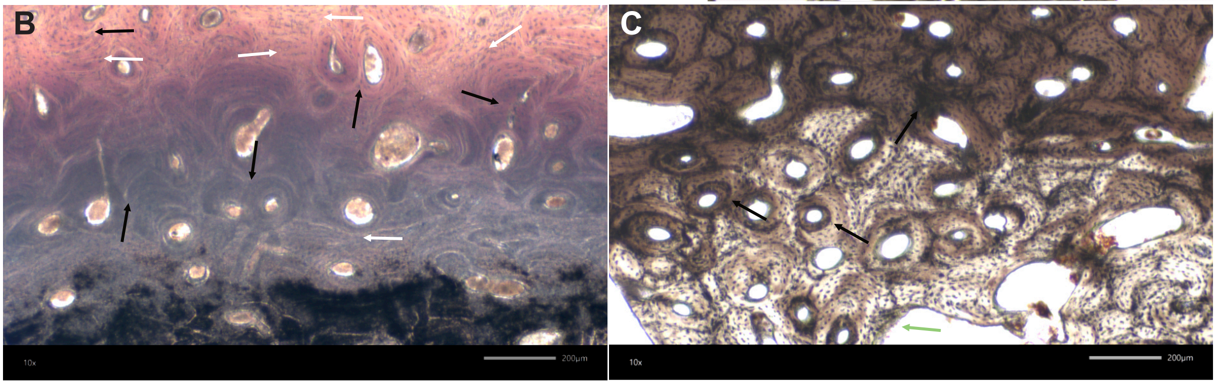

Figure 17.5-6: Histomorphology: age indicators, fire impact, and bioerosion.

INDIVIDUAL 5, no fire impact. A-C: left parietal, vertical section. **A**: Lamina externa and diploe, plain light, 5.6x magnification. **B**: Cortical layer lamina interna, polarized light, x10 magnification. **C**: Cortical layer lamina interna, plain light, x10 magnification.

Histomorphological features: OHI of 1-2, poorly preserved microstructure; few osteons observable, poor collagen and lamellar preservation (B, green arrows). Higher image resolution was not achievable. Few postmortem damage on bone surfaces. Not suitable for evaluating age.

Bioerosion: Massive histotaphonomical damage by microbial attack with microscopic focal deconstruction. B: Suspected Wedl (Wt), budded/longitudinal (BLt) and lammelate tunneling (Lt). C:Suspected Wedl and budded/longitudinal tunneling.

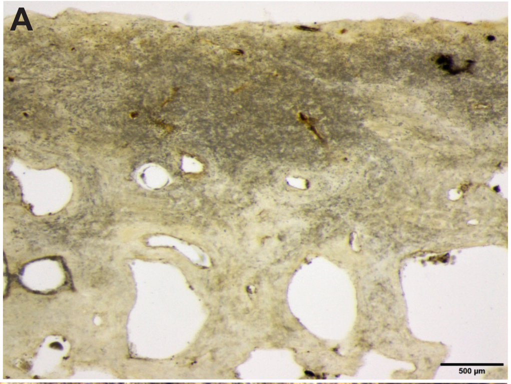

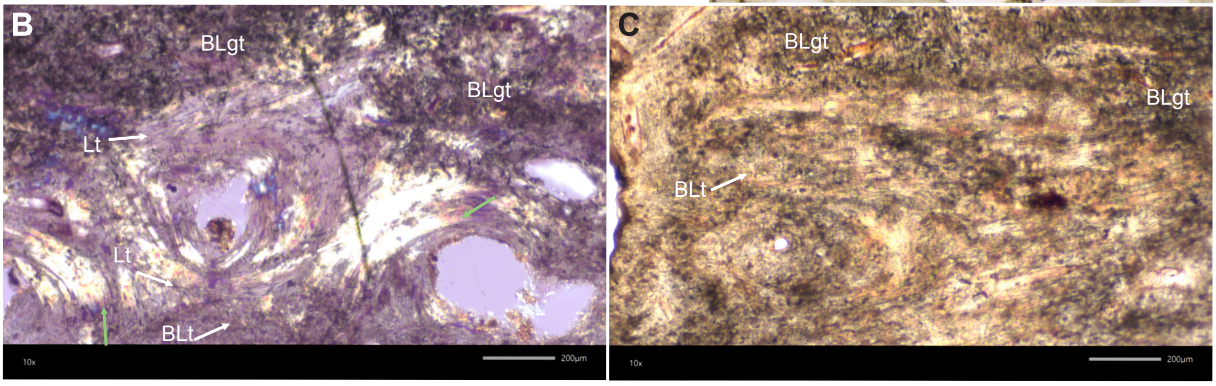

INDIVIDUAL 7, no fire impact. A-C: occipital, vertical section nuchal plane. **A**: Full section, plain light, 5.6x magnification. **B**: Cortical layer lamina externa, polarized light, x10 magnification. **C**: Cortical layer lamina interna, plain light, x10 magnification.

Histomorphological features: OHI of 2-3, moderately preserved microstructure; osteons with osteocytes observable, collagen and lamellar preservation only partly. Few postmortem damage on bone surfaces. Osteon density and presence of different osteon generations (A, black arrows) are indicative for an adult individual.

Bioerosion: Moderate histotaphonomical damage by microbial attack with microscopic focal deconstruction. B, C: Suspected Wedl (Wt), budded tunneling (Bt).

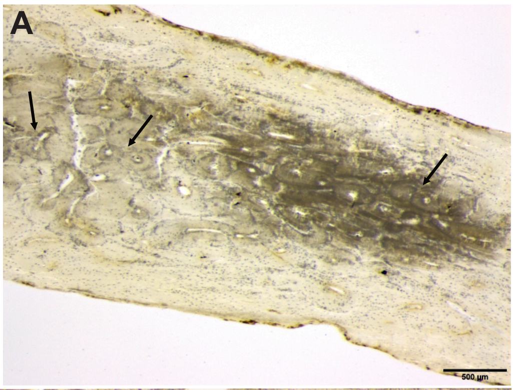

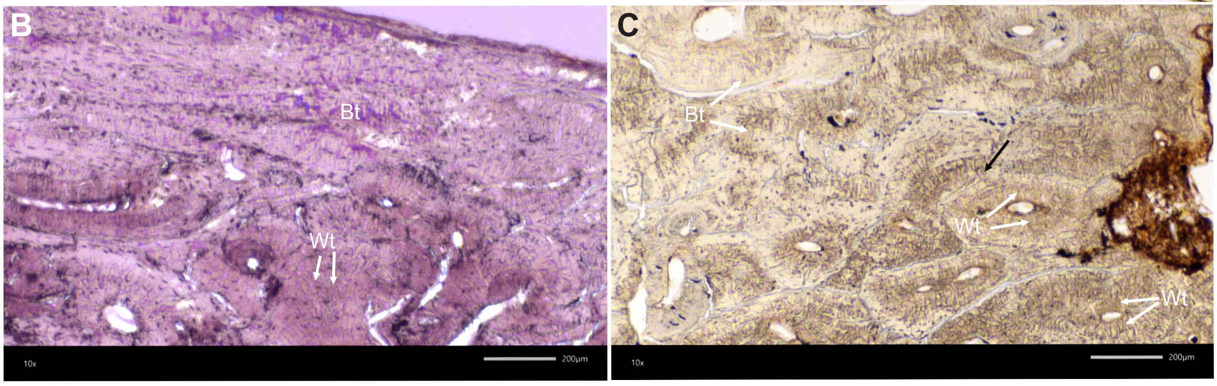

Figure 17.7: Histomorphology: age indicators, fire impact, and bioerosion.

Isolated right humerus, no fire impact. A-C: distal epiphysis, above olecranon fossa, transversal section. **A**: Periosteal and inner cortical bone, plain light, 5.6x magnification. **B**: As A, polarized light, x10 magnification. **C**: Inner cortical bone, polarized light, x10 magnification.

Histomorphological features: OHI of 3, moderately preserved microstructure; osteons, osteocytes, collagen and lamellar bone observable. Few postmortem damage on bone surfaces. Osteon density and presence of different osteon generations (A, black arrows) are indicative for an adult age-at-death.

Bioerosion: Strong histotaphonomical damage by microbial attack with microscopic focal deconstruction. **B, C**: Suspected lammelate (Lt), budded and longitudinal tunneling (Bt, Lgt); the latter are often indistinguishable (BLgt).

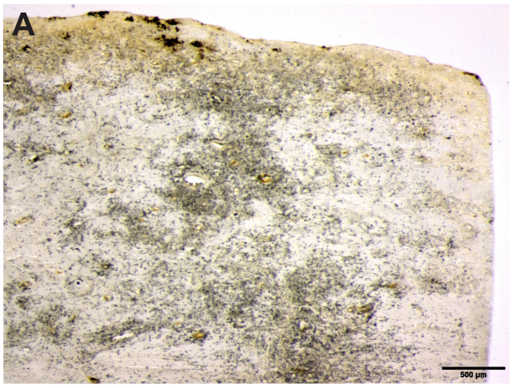

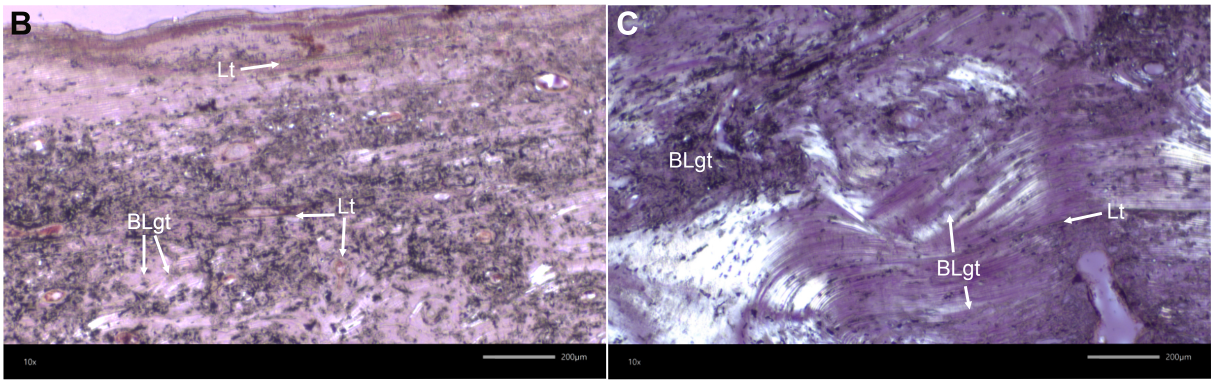

Figure 18: Fragmentation morphology.

Left distal humerus (1), right proximal ulna (2), and right distal radius (3) showing V-shaped fractures of the diaphyses. Jagged, irregular outlines with chipped surface, atypical for completely fresh bone (arrows), but too smooth for late post-mortem breakage of dry bone.

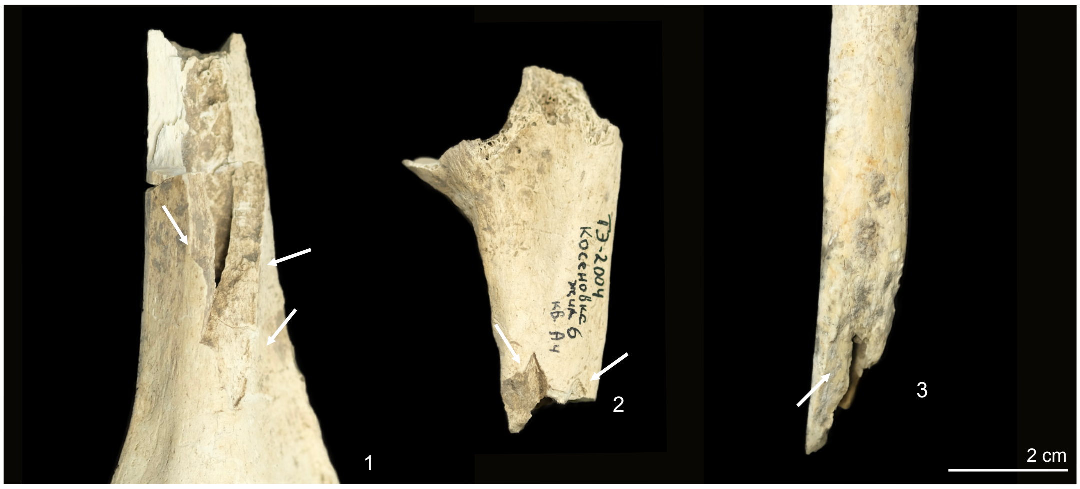

Supplement: S1 Appendix — Skeletal inventory and microscopy photos with descriptions. (PDF) [file pone.0289769.s001.pdf]
