# Supplementary material for: Life and death in Trypillia times: Interdisciplinary analyses of the unique human remains from the settlement of Kosenivka, Ukraine (3700–3600 BCE)
Source: PLoS One. 2024 Dec 11;19(12):e0289769. doi: 10.1371/journal.pone.0289769 (PMC11633957; doi:10.1371/journal.pone.0289769)

Figure 18: Collagen yield vs. ^{14}C result of human and faunal samples from the Kosenivka house 6 measured at the Radiocarbon Laboratory, Poznan (see S1 Tables).

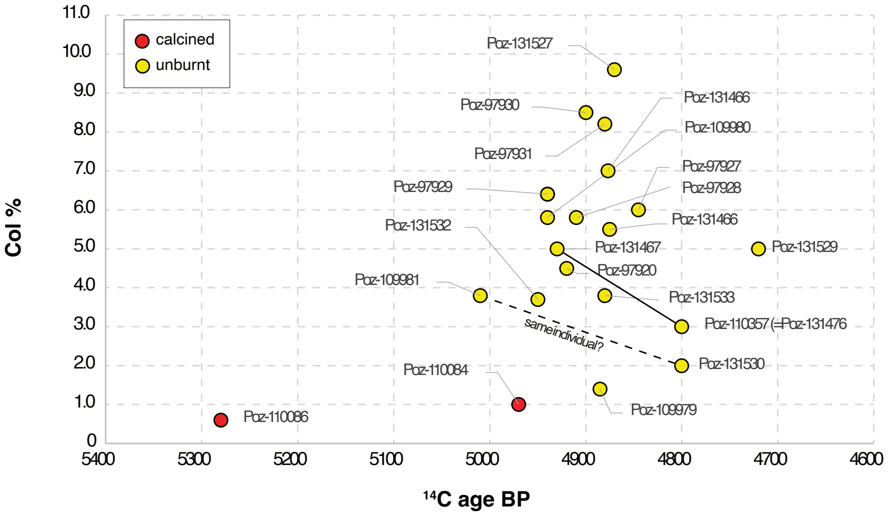

Figure 19: Calibration curve of all radiocarbon dates obtained for Kosenivka (n=26) using OxCal v. 4.4.

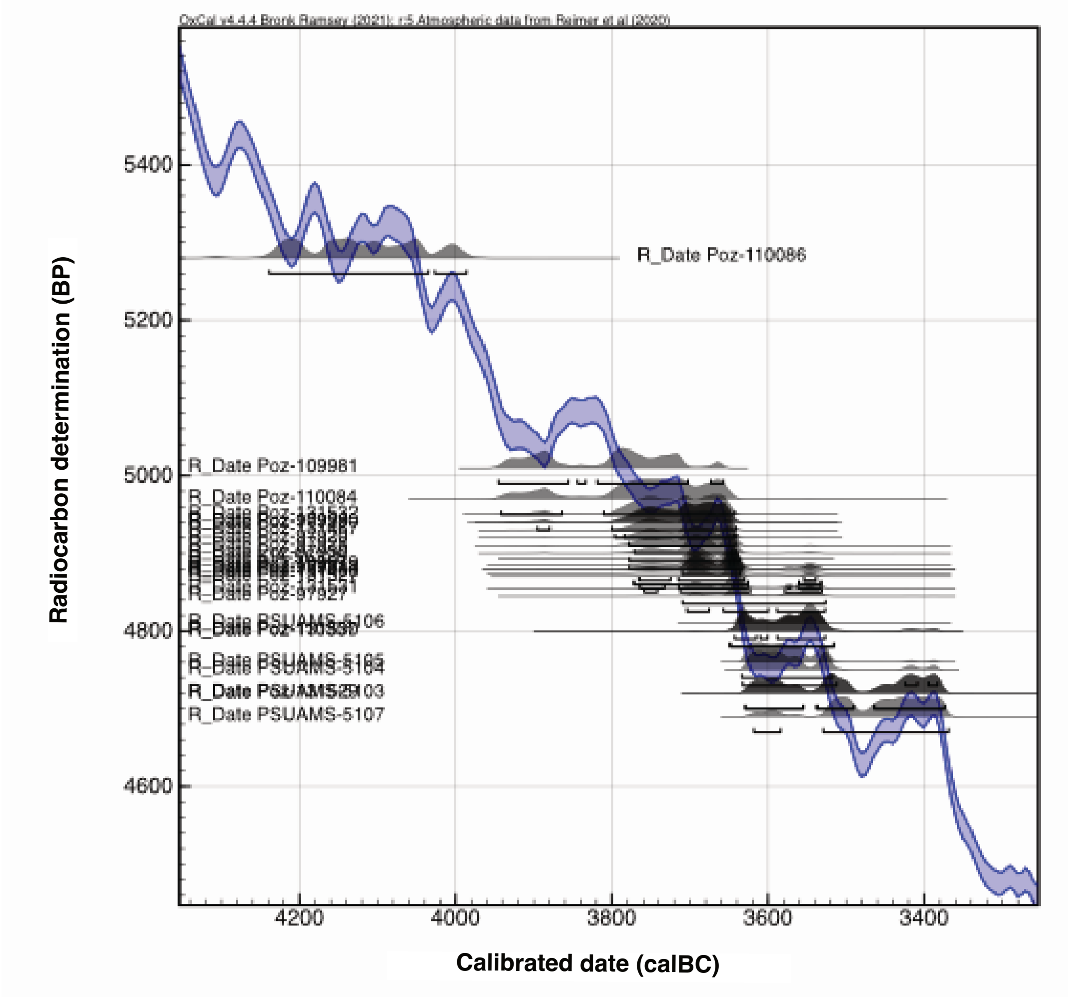

Figure 20: Curve plot Bayesian modelling of radiocarbon dates with context information obtained for Kosenivka (n=24, excluded is Poz-11086), using OxCal v. 4.4. For original code see *S3 Tables*.

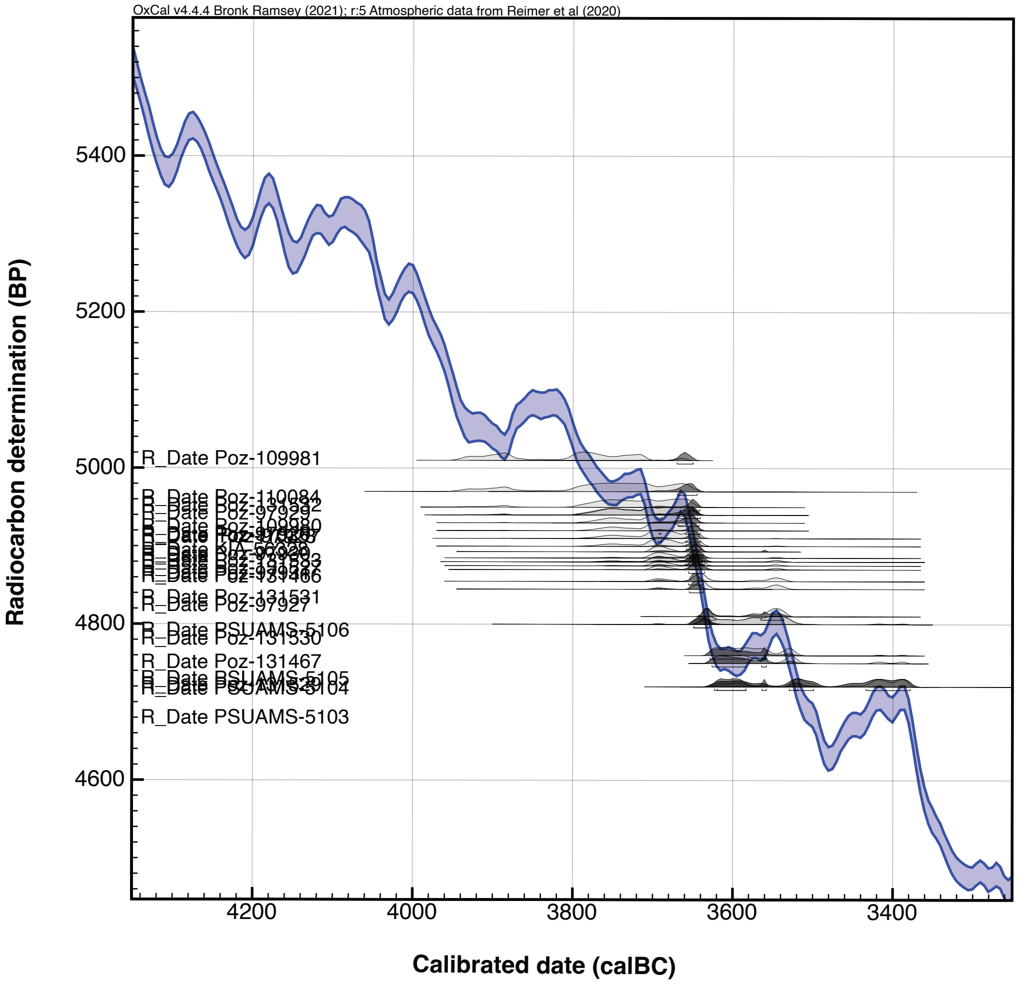

Supplement: S2 Appendix — Sample quality, OxCal curves unmodelled and modelled data. (PDF) [file pone.0289769.s002.pdf]
